# Confidence and self-attribution bias in an artificial stock market

**DOI:** 10.1371/journal.pone.0172258

**Published:** 2017-02-23

**Authors:** Mario A. Bertella, Felipe R. Pires, Henio H. A. Rego, Jonathas N. Silva, Irena Vodenska, H. Eugene Stanley

**Affiliations:** 1 Department of Economics, Sao Paulo State University (UNESP), Sao Paulo, Brazil; 2 Sao Paulo Metropolitan Company, Sao Paulo, Brazil; 3 Federal Institute of Education, Science and Technology, Maranhao, Brazil; 4 Metropolitan College, Boston University, Boston, Massachusetts, United States of America; 5 Center for Polymer Studies and Department of Physics, Boston University, Boston, Massachusetts, United States of America; East China University of Science and Technology, CHINA

## Abstract

Using an agent-based model we examine the dynamics of stock price fluctuations and their rates of return in an artificial financial market composed of fundamentalist and chartist agents with and without confidence. We find that chartist agents who are confident generate higher price and rate of return volatilities than those who are not. We also find that kurtosis and skewness are lower in our simulation study of agents who are not confident. We show that the stock price and confidence index—both generated by our model—are cointegrated and that stock price affects confidence index but confidence index does not affect stock price. We next compare the results of our model with the S&P 500 index and its respective stock market confidence index using cointegration and Granger tests. As in our model, we find that stock prices drive their respective confidence indices, but that the opposite relationship, i.e., the assumption that confidence indices drive stock prices, is not significant.

## Introduction

In recent decades the efficient market hypothesis (EMH) has been generally assumed to be true in finance [1]. In his classic paper, Fama [2] defined an efficient financial market as one in which asset prices always fully reflect available information. The EMH is based on three arguments, (i) that investors are rational, perfectly consistent and coherent as they critically examine their options, and possess enormous computational power, (ii) that some investors are irrational but because their actions are random they cancel themselves out and do not affect asset prices, and (iii) that when irrational investors begin to act in concert they are stopped by rational arbitrageurs who eliminate their influence on asset prices [1].

In the first decade after its development in the 1960s, the EMH became unanimously accepted, both among theoreticians and those working empirically. Jensen, one of the creators of the EMH, stated “there is no other proposition in economics which has more solid empirical evidence supporting it than the efficient market hypothesis” [3].

In the years that followed, this hypothesis began to be questioned not only from a theoretical but also from an empirical point of view. First, to bluntly state that people in general and investors in particular are totally rational is problematic. According to Fisher Black [4], investors trade on noise instead of on information, but this statement is overly general because investor behavior is often simultaneously irrational and highly systematic.

Tversky and Kahneman [5] point out that trader actions can indicate a departure from the conventional rational decision model in several fundamental areas, including their attitudes towards risk, their mental accounting, and when they exhibit overconfidence. Awareness of these psychological factors and of the reality that arbitrage is limited has produced a new approach to the study of financial markets: behavioral finance (BF).

Although conventional financial market models based on such hypotheses as rational selection and market efficiency are elegant, none has been able to explain such basic empirical characteristics or “anomalies” in real-world financial markets as excessive transaction volume or price volatility. Thus financial markets have become one of the most active areas within which researchers using agent-based models attempt to understand regularities found in financial data. One of the first studies of this type was conducted by Arthur et al. [6], who developed a dynamic theory of asset pricing that was based on heterogeneous investors who update their price expectations individually and inductively using classification systems.

Agent-based computational models treat economies as systems made up of independent agents who interact with each other according to a set of rules. The initial market conditions are specified and the economy is allowed to evolve over time as the constituent agents repeatedly interact. The goal is to investigate the relationship between market prices and information.

With respect to price formation, which is critical, such models can be classified under four categories according to LeBaron [7]. The first type uses a slow price adjustment, in which the market is always in fact in disequilibrium. An example of this category of models is Day and Huang [8]. A second market mechanism is to set the equilibrium at each period of time numerically or analytically (the latter method assuming simplifying hypotheses). Examples of this kind of market adjustment are Arthur et al. [6], Brock and Hommes [9], and more recently Xu et al. [10], among others. A more realistic and perhaps a more important mechanism is the simulation of an order book, in which the agents define offers to buy and sell stock. The orders are then crossed in conformity with some defined procedure, like in Farmer et al. [11], and Zhou et al. [12]. In Farmer et al. [11], a model is tested in which the agent’s rationality is eliminated almost completely. The model assumes the agents place buy and sell orders randomly, subject to constraints imposed by the prices. The authors demonstrate that such approach is able to replicate many characteristics of the price history dataset, that is, this paper helps to understand which empirical regularities may be the fruit of only the institutions, and which may be the result of the agents’ learning. On the other hand, Zhou et al. [12] study the order flow of Shenzhen (China) Stock Exchange for the year 2003. Among other important results, it is observed that random strategies showed a much better performance than real strategies both for winners and losers, what seems to corroborate the model by Farmer et al. [11]. Matching is another kind of adjustment mechanism, where the agents meet at random and if it is convenient for them, they trade with one another. This mechanism may be suitable for situations where formal trading markets have not been established yet. An example of this type of model can be found in Beltratti and Margarita [13].

Agent-based models can contribute significantly to the study of financial behavior by computationally analyzing these psychological characteristics. Note that agent-based models applied to finance are behavioral models themselves because the agents are limited rationally and usually follow rules that are either preset or learned through experience. Most of the models created thus far depart from the behavioral finance approach, however, in that they assume that the agents exhibit conventional preferences.

Our goal here is to create an agent-based model in which the agents exhibit confidence in their decision making, in accordance with the behavioral finance approach, and we assume that the level of agent confidence evolves during the simulation time. According to Odean [14], the overconfidence of successful agents can be reinforced by a self-attribution bias, i.e., when they believe their trading success is the result of their own ability. A small number of papers in the literature incorporate psychological biases into the agents, among them the studies by Takahashi and Terano [15], Lovric [16], and Bertella et al. [17]. Takahashi and Terano [15] use the Bayes error correction model, Lovric [16] the model by Levy, Levy, and Solomon [18], and Bertella et al. [17] a study by Arthur et al. [6]. Our study is similar to that by Bertella et al. [17], but it differs in the way we model confidence and how we verify the robustness of our model.

The study is organized as follows: section one describes the model framework, section two how agent expectation is determined, section three the behavioral bias that affects agent decisionmaking, section four the econometric analysis of our model, comparing it with the data from the S&P 500 index and its confidence levels and section five presents some final considerations.

## Model

Our model is based on a study by Bertella et al. [17], and is composed of N agents who decide whether to invest in a risky asset (e.g., a stock) or in one that is risk-free (e.g., a US Treasury security).

The dividend paid by the stock per time unit, based on studies by Arthur et al. [6] and LeBaron et al. [19], is

$$\begin{array}{c} d_{t}=\bar{d}+\rho \;\left(d_{t-1}+\bar{d}\right)+\epsilon_{t} \end{array}$$ (1)

where $\bar{d}$ is the dividend base, *ϵ*_*t*_ has a normal distribution with mean 0 and finite variance *σ*^2^, and 0 < *ρ* < 1. The utility function is

$$\begin{array}{c} U\left(W_{i,t}\right)=-e^{-\lambda w_{i,t}} \end{array}$$ (2)

where *W*_*i*,*t*_ is the wealth of agent *i* at time *t* and *λ* is the level of risk aversion. The maximization of the expected utility is subject to the budget constraint

$$\begin{array}{c} W_{i,t}=x_{i,t}\left(p_{t}+d_{t}\right)+\left(1+r\right)\left(W_{i,t-1}-p_{t}x_{i,t}\right) \end{array}$$ (3)

where *W*_*i*,*t*_ represents the wealth of agent *i* at time *t*, *x*_*i*,*t*_ represents the quantity of stocks ordered by agent *i*, *p*_*t*_ and *d*_*t*_ are the price and stock dividend respectively at time *t*, and *r* corresponds to the interest rate for the risk-free asset, considered constant over time.

The optimal quantity of stocks ordered by each agent *x*_*i*,*t*_ is

$$\begin{array}{c} x_{i,t}=\frac{E_{i,t}\left(p_{t+1}+d_{t+1}\right)-p_{t}\left(1+r\right)}{\lambda \sigma_{i,t,p+d}^{2}} \end{array}$$ (4)

where $\sigma_{i,t,p+d}^{2}$ is the perceived variance of the returns, described by

$$\begin{array}{c} \sigma_{i,t,p+d}^{2}=\left(1-\theta \right)\sigma_{i,t-1,p+d}^{2}+\theta {\left[p_{t}+d_{t}-E_{i,t-1}\left(p_{t}+d_{t}\right)\right]}^{2} \end{array}$$ (5)

in which parameter *θ* determines the weight placed on the most recent square error as opposed to the weight placed on past square errors.

The market price is determined by the difference between the quantity of stocks ordered by agent *i* at *t* and the quantity at a previous time *t* − 1. If this difference is positive or zero, the number of stocks that agent *i* will buy at *t* (*b*_*i*,*t*_) will be the difference itself, and the number of stocks that the agent will sell at the same time *t* (*o*_*i*,*t*_) will be zero. This situation will reverse when the difference is negative. By adding the contribution of *b*_*i*,*t*_ and *o*_*i*,*t*_ together for all agents, we can determine the total quantity demanded, *B*_*t*_, and supplied, *O*_*t*_. Thus, according to Farmer and Joshi [20], the stock price at time *t* is

$$\begin{array}{c} p_{t}=p_{t-1}{\text{e}}^{\frac{B_{t}-O_{t}}{\beta }} \end{array}$$ (6)

where parameter *β* eases price fluctuations in the market.

The rate of return at time *t* can then be calculated using

$$\begin{array}{c} H_{t}=\frac{p_{t}-p_{t-1}+d_{t}}{p_{t-1}} \end{array}$$ (7)

### Formation of expectations and trading strategies

For the formation of expectations regarding the price and future dividend of the stock traded, *E*_*i*,*t*_(*p*_*t*+1_ + *d*_*t*+1_), the fundamentalists assume certain rules based on the dividend at time *t* and therefore estimate that growth will be at a constant rate *g*, i.e.,

$$\begin{array}{c} E\left(d_{t+1}\right)=d_{t}\left(1+g\right) \end{array}$$ (8)

and

$$\begin{array}{c} E\left(p_{t+1}\right)=\frac{d_{t}\left(1+g\right)}{k-g} \end{array}$$ (9)

in which *k* refers to the discount rate of the flow of future dividends. On the other hand, the chartists estimate that prices are inertial, that is, if the recent price has increased the future price will also increase, and vice-versa. Thus based on a study by Takahashi and Terano [15], our expectations of price and future dividends will be

$$\begin{array}{c} E\left(p_{t+1}\right)=p_{t-1}{\left(1+a_{t-m}\right)}^{2} \end{array}$$ (10)

and

$$\begin{array}{c} E\left(d_{t+1}\right)=d_{t-1}\left(1+a_{t-m}\right) \end{array}$$ (11)

in which term $a_{t-m}=\frac{1}{m}\sum_{m=1}^{m}\left(\frac{p_{t-1}}{p_{t-1-m}}-1\right)$ is associated with memory length, which can be of one, five, or ten units of time (m = 1, 5, and 10, respectively). Fundamentalists believe stock price converges to fundamental value and they use a dividend discount model to estimate it. Chartists or technical traders are trend predictors which use past information to predict future prices.

We carry our simulation for 100 agents, arbitrarily distributed between chartists and fundamentalists, who can—at each period of time—order and sell (short) up to a maximum of five stocks.

### Confidence and self-attribution bias

According to Barberis and Thaler [21], behavioral finance studies can be divided into two categories:

1. those that show that arbitrage operations are usually unable to keep stock prices attached to their fundamental values; and
2. those that demonstrate that agents commit systematic errors when facing uncertainty and deviate from conventional assumptions.

The first category of study demonstrates that arbitrage operations are not perfect. The second makes it clear that psychology influences a family’s decisions about consumption and investment. According to Kahneman and Riepe [22], financial decisions in uncertain environments are based on established rules and intuition. Thus either an excess or deficit of confidence can affect the actions of an economic agent and lead to irrational trading decisions.

In our study we use the perceived variance of stock returns described by Eq (5) and create a confidence coefficient that, when multiplied by the perceived variance of returns, characterizes its under—or over—estimation,

$$\begin{array}{c} {\hat{\sigma }}_{i,t,p+d}^{2}=oc_{i,t}\times \sigma_{i,t,p+d}^{2} \end{array}$$ (12)

where coefficient *oc* represents the level of agent confidence. When *oc* = 1, the agent has a neutral level of confidence and the variance of the stock return is not underestimated. When *oc* > 1, the agent lacks confidence and the variance of the stock return is overestimated. When 0 ⩽ *oc* < 1, the agent is overconfident and the variance of the stock return is underestimated, i.e., agents strongly believe in the validity of their stock return predictions.

We assume that the level of agent confidence evolves during the simulation time. As mentioned above, the overconfidence of successful agents can be further strengthened by a self-attribution bias. The level of agent confidence is updated based on the success of their predictions. We carry out this updating by first mapping confidence coefficient *oc* from interval *oc* ∈ [0, ∞[ into a more convenient interval, *C* ∈ [0, 1]. Thus, as described by Lovric [16], we use a transformation function *T*,

$$\begin{array}{c} T\left(oc_{i,t}\right)=1-2^{{\left(-oc_{i,t}\right)}^{1/4}}=C_{i,t} \end{array}$$ (13)

The transformation function *T*(…) is defined so that the neutral level of confidence (*oc*_*i*,*t*_ = 1) can be mapped at the mean point of the transformation function (*C*_*i*,*t*_ = 0.5). After the level of agent confidence is transformed into interval *C* ∈ [0, 1], the levels are updated according to

$$\begin{array}{c} \begin{array}{c} \text{If}\;|E_{i,t-1}\left(p_{t}+d_{t}\right)-p_{t}-d_{t}|<2\cdot oc_{i,t}\cdot \sigma_{i,t,p+d}\text{,}\\ \;\text{then}\;C_{i,t+1}=C_{i,t}\cdot \bar{a}\\ \;\text{otherwise}\;C_{i,t+1}=C_{i,t}\cdot \bar{b} \end{array} \end{array}$$ (14)

where *σ*_*i*,*t*,*p*+*d*_ corresponds to the perceived standard deviation of the stock return. If the difference between the expected stock return and the actual return is within the interval of confidence defined by the agents, then the level of confidence will be decreased by parameter $\bar{a}$. If it is not, the agents are less confident and *C*_*i*,*t*_ is multiplied by parameter $\bar{b}$.

Note that $\bar{b}>1$ and $0<\bar{a}<1$. It is possible that the updating of the level of agent confidence is biased. For example, the increase in confidence level for good predictions can be greater than the decrease in confidence level for bad predictions. An example of a non-biased self-attribution bias occurs when $1-\bar{a}=\bar{b}-1$, where $\bar{a}=0.99$ and $\bar{b}=1.01$.

After the level of agent confidence is updated, *C*_*i*,*t*+1_ it is mapped at the original interval [0, ∞[ using the inverse transformation function as described by Lovric [16],

$$\begin{array}{c} oc_{i,t+1}=T^{-1}\left(C_{i,t+1}\right)={\left[\frac{\ln(1-C_{i,t+1})}{\ln0.5}\right]}^{4} \end{array}$$ (15)

## Results and discussion

This section describes the computer simulations and discusses the results. The simulations are carried out as follows:

1. In the first simulation we focus on the behavioral heterogeneity of agents with a neutral level of confidence in a market composed of 25 fundamentalist agents, 25 chartist agents with *m* = 1, 25 chartist agents with *m* = 5, and 25 chartist agents with *m* = 10.
2. In the second simulation we use the same market configuration but vary the levels of chartist agent confidence. The simulation and its descriptive statistics in which all agents are fundamentalists (reference case) are shown in the S1 Appendix file (A1 Table, A2 Table, A1 Fig and A2 Fig), as well as the initial values of the parameters in the simulations.

### Heterogeneous agents with neutral confidence

**Fig 1:**
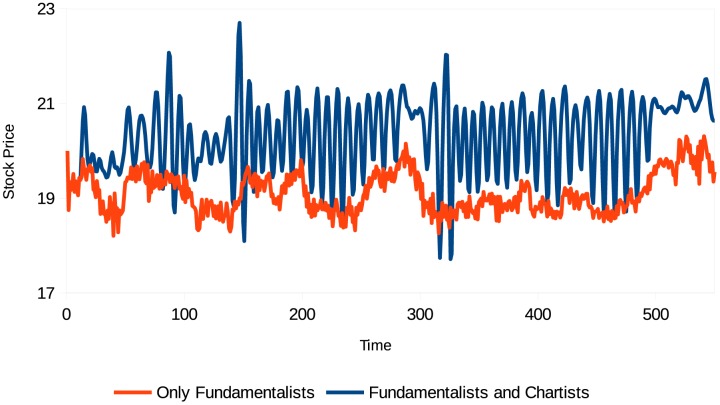
Evolution of the stock price (different types of agents). Source: Own creation.

**Fig 2:**
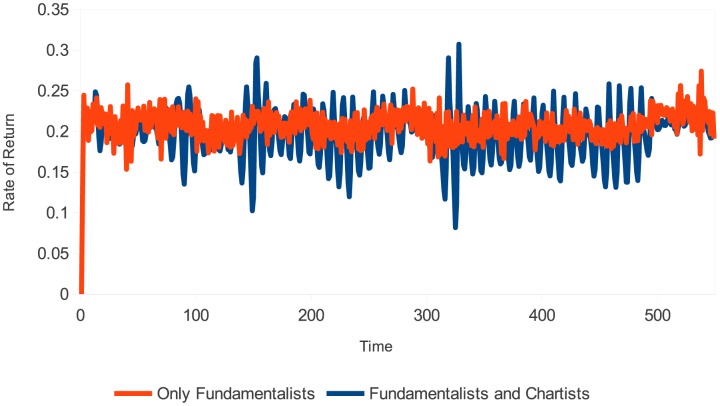
Evolution of the stock rate of return (different types of agents). Source: Own creation.

In this simulation, agents can adopt different trading strategies. The market is composed of fundamentalist and chartist agents, with different memory lengths (*m* = 1, *m* = 5, and *m* = 10). The results are shown in Figs 1 and 2.

Fig 1 compares the evolution of the stock price with the reference case (in which there are only fundamentalists). The evolution pattern of the stock price differs entirely from that of the reference case. Thus the presence of behavioral heterogeneity in the market may explain the excess volatility and systematic deviations of the asset prices from their fundamental values. Note that there are periods when the stock price is sustainably higher than the reference price, periods when the market is volatile, and periods of extreme volatility, which are characteristic of market crashes.

**Fig 3:**
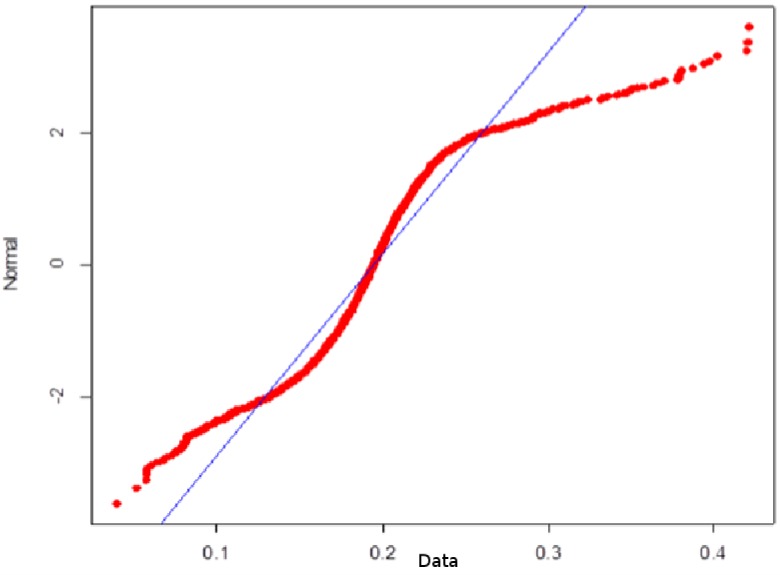
Shapiro-Wilk Normality Test stock rate of return (different types of agents). Source: Own creation.

Fig 2 shows the evolution of the stock rate of return during the simulation time, which confirms the presence of excess volatility in the market. Excess volatility occurs at periods when the dividend value generated by the stock breaks the trend heretofore maintained. The chartist agents do not expect this break because they do not know the value of the dividend generated in the prior period. The greater the number of chartist agents in the market, the greater the impact of their actions and the higher the market volatility. Statistics for this simulation, as well as the normality test for the rate of return, are shown in Table 1 and Fig 3, respectively.

**Table 1:** Descriptive statistics (different types of agents). Source: Own creation.

	Stock Price	Return Rate
**Mean**	20.5618	0.1946
**Median**	20.7289	0.1945
**St. Deviation**	0.8131	0.0305
**Kurtosis**	5.3023	8.8112
**Skewness**	−1.3694	0.9167

Note that the mean and median values of the return rate are lower than the values in the reference case, but the standard deviation and kurtosis values are higher, which indicates a substantial increase in volatility, the presence of heavy tails, and a considerable discrepancy in the normal distribution (see Fig 3). All of these characteristics are commonly found in financial series and may be the result of behavioral heterogeneity in the financial market.

### Heterogeneous agents with different levels of confidence

**Fig 4:**
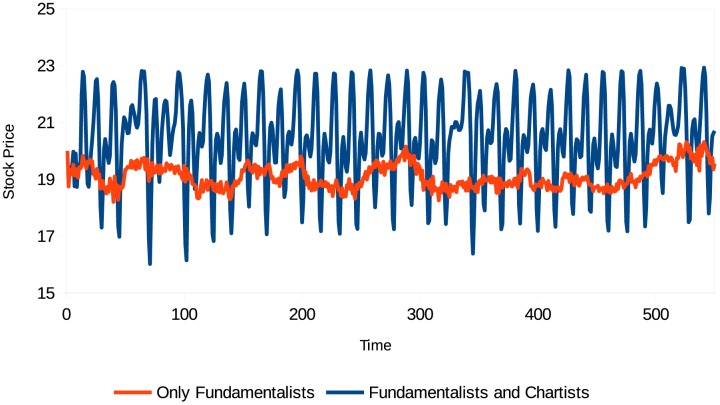
Stock price (different types of agents with confidence). Source: Own creation.

**Fig 5:**
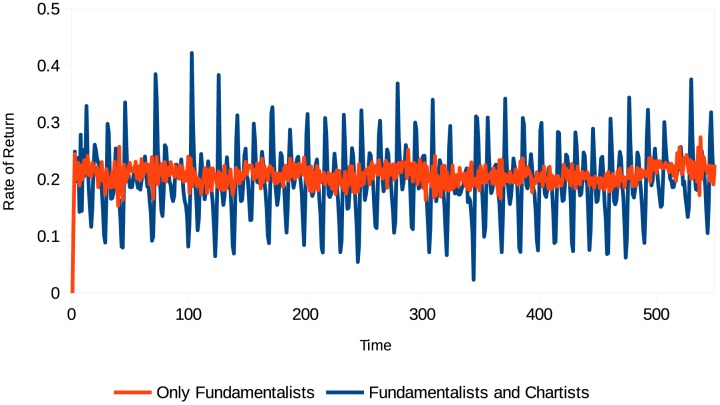
Rate of return (different types of agents with confidence). Source: Own creation.

**Fig 6:**
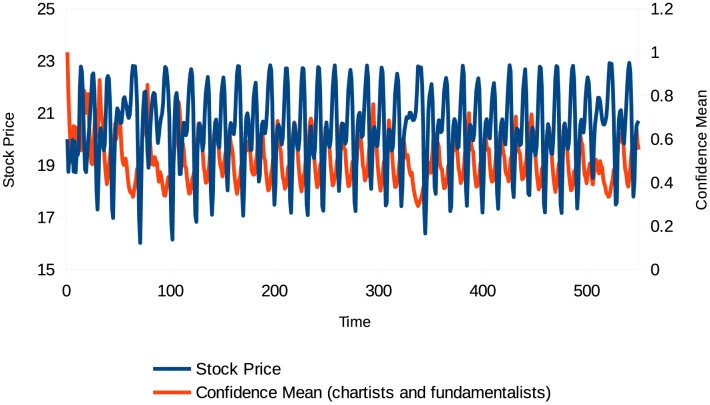
Level of confidence (different types of agents with confidence). Source: Own creation.

The next simulation focuses on the interaction between different types of agents in the market and allows their confidence levels to evolve during the simulation. The market is composed of 25 fundamentalist agents not influenced by confidence and 75 chartist agents influenced by confidence and divided equally according to their memory of analysis. Figs 4–6 show the results.

This simulation takes into account behavioral heterogeneity, but also the effect of the changing levels of agent confidence. This additional factor allows us to analyze and explain additional characteristics. Figs 4–6 show that the periods when assets are sharply overvalued coincide with those when agent confidence level is high, and the periods when prices fall coincide with those when agent confidence level is low. Note that in this case the volatility is also much higher than when agents have a “neutral” confidence level.

**Fig 7:**
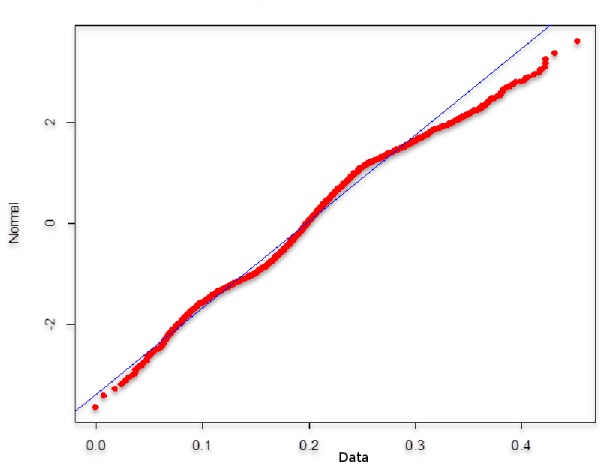
Shapiro-Wilk Normality Test stock rate of return (different types of agents with confidence). Source: Own creation.

Table 2 shows the descriptive statistics and the Fig 7 reveals the normality test for the stock rate of return for this simulation. Note how the standard deviation of the return rate is higher than in the previous case, indicating an increase in volatility. An excess of confidence intensifies volatility, and kurtosis and skewness are less than in the previous case (which had heterogeneous agents with a neutral confidence level), but the distribution of the rates of return remains far from normal.

**Table 2:** Descriptive statistics (different types of agents with confidence). Source: Own creation.

	Stock Price	Return Rate
**Mean**	20.4605	0.1972
**Median**	20.5626	0.1974
**St. Deviation**	1.4671	0.0578
**Kurtosis**	−0.1429	1.1202
**Skewness**	−0.5265	0.2280

## Econometric analysis

We next measure the robustness of our model by comparing its results with actual data. We first analyze two variables produced by the model, confidence and price, to determine whether confidence determines price or price determines confidence. We then compare the results with actual S&P 500 index data and with confidence indices calculated for this stock market to determine whether real-world confidence levels determine stock prices or stock prices determine confidence levels.

### Unit root tests

The hypotheses were first tested for the time series describing random walks: (i) the confidence index *C*_*t*_ and (ii) the stock price *P*_*t*_, using the following unit root tests:

1. Ljung-Box Test – LB (Ljung & Box, 1978) [23];
2. Phillips-Perron Test – PP (Phillips & Perron, 1988) [24];
3. Augmented Dickey-Fuller Test – ADF (Dickey & Fuller, 1979; and MacKinnon, 1991) [25, 26];
4. Kwiatkowski-Phillips-Schmidt-Shin – KPSS (Kwiatkowski et al. 1992) [27].

The adopted econometric procedure tested the following modified pair of hypotheses:

1. $H_{0}^{\prime }$: series *C*_*t*_ and *P*_*t*_ are nonstationary
2. $H_{1}^{\prime }$: series *C*_*t*_ and *P*_*t*_ are stationary

**Fig 8:**
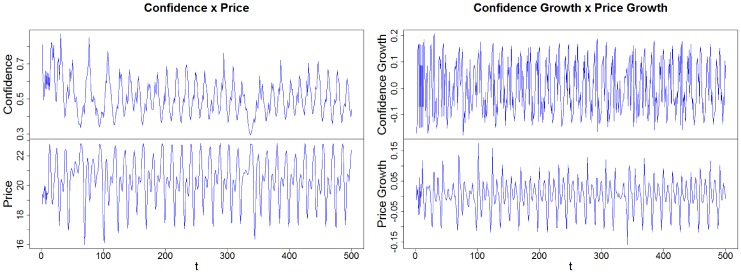
Time series illustrating (on the left) the confidence index and the stock price and (on the right) their respective growth rates. Source: Own creation.

Fig 8 shows the time evolution containing a sub-set of 500 values for the confidence index *C*_*t*_ and the stock price *P*_*t*_, and their respective growth rate, suggesting a non-stationarity behavior in all cases.

All four unit root tests corroborate the results shown in Fig 8 and use as a base a significance level of 5% (p-value < 0.05), as shown by the values in Table 3.

**Table 3:** Unit root test.

	Indexes		Growth Rate	
	Confidence	Price	Confidence	Price
**LB Test (p < 0.05)**	p = 0.0 (TRUE)	p = 0.0 (TRUE)	p = 0.0 (TRUE)	p = 0.0 (TRUE)
**PP Test (p < 0.05)**	p = 0.01 (TRUE)	p = 0.01 (TRUE)	p = 0.01 (TRUE)	p = 0.01 (TRUE)
**ADF Test (p < 0.05)**	p = 0.01 (TRUE)	p = 0.01 (TRUE))	p = 0.01 (TRUE)	p = 0.01 (TRUE))
**KPSS Test (p > 0.05)**	p = 0.01 (TRUE)	p = 0.1 (TRUE)	p = 0.01 (TRUE)	p = 0.1 (TRUE)
**ADF *τ* critical values** **1% 5% 10%** **-2.58 -1.95 -1.62**	*τ* = -28.4477 (TRUE)	*τ* = -28.1368 (TRUE)	*τ* = -33.3431 (TRUE)	*τ* = -54.1440 (TRUE)
**KPSS *τ* critical values** **1% 2.5% 5% 10%** **0.216 0.176 0.146 0.119**	*τ* = 0.1481 (TRUE)	*τ* = 0.0406 (FALSE)	*τ* = 0.2161 (TRUE)	*τ* = 0.1511 (TRUE)

Thus the results for stock price, its growth rate, confidence index and its growth rate show that we cannot reject the null hypothesis and thus all the series have a unit root and are non-stationary.

### Cointegration and Granger tests

The random walk analyses carried out in the previous section confirm the non-stationarity of the series analyzed individually. We now test the series for cointegration in order to determine whether there is a long-range temporal relationship between the two variables, price and confidence, and their growth rates. We test two hypotheses:

1. *H*_0_: series *C*_*t*_ and *P*_*t*_ are not cointegrated
2. *H*_1_: series *C*_*t*_ and *P*_*t*_ are cointegrated

**Fig 9:**
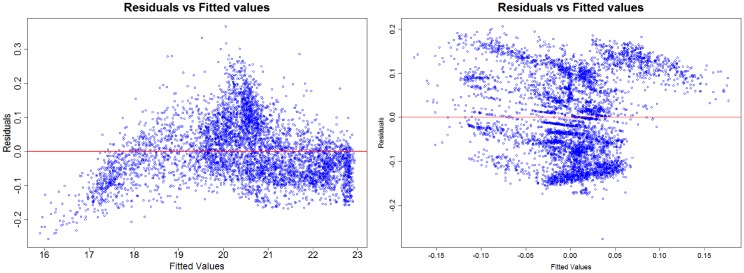
Residuals from the regressions (on the left) “confidence vs price” and (on the right) “confidence vs price” formulas. Source: Own creation.

Following Engle and Granger (1987) [28], we test whether the series cointegrate by first conducting an ordinary least squares (OLS) regression to estimate a time series. Fig 9 shows the residuals obtained through this regression for the data used, which in this case are stock prices.

First, to test the cointegration for the price index with respect to confidence index, we use the most negative Dickey-Fuller (DF) value to select which regression formula we will apply. When we test the residuals of the regression “confidence vs price” for the existence of a unit root we get a value of −27.366 for statistic t, with p < 2.2e-16 (and a value of -15.812 for the formula “price vs confidence”), and find that we can reject the null hypothesis. Thus we assume that the residual is stationary, and that we cannot reject the hypothesis that the series are cointegrated, suggesting that there is a relationship between confidence and the price index.

We obtain a similar result for the growth rate of the indices. Since the DF value for the regression formula “growth of confidence vs growth of price” is −62.2409, and −42.0239 for its opposite, we choose “growth of confidence vs growth of price” to be the dependent variable and find that we can also, at a 90% confidence level (p-value is 10%), reject the null hypotesis.

**Fig 10:**
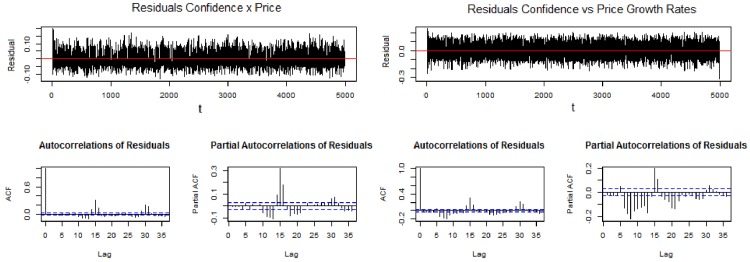
Autocorrelation function of residuals from the regressions (on the left) “confidence vs price” and (on the right) “confidence vs price” formulas. Source: Own creation.

Fig 10 shows the autocorrelation function (ACF) of residuals for the test by Ljung–Box (1978), and Table 4 shows the results of autocorrelation tests for residuals, revealing a correlation between confidence and price, thus supporting the hypothesis that the series are cointegrated.

**Table 4:** Johansen test for confidence x stock price.

	Value test for the regression formula:		Level of confidence		
	“confidence vs price”	“growth of confidence vs growth of price”	10%	5%	1%
*r* = 0	754.49	796.67	6.50	8.18	11.65
*r* ≤ 1	6269.19	4019.59	15.66	17.95	23.52

To confirm the cointegration among the series we use the Johansen test [29] to estimate the confidence level rank of a series or a set of series to test for the null hypothesis of *r* = 0 (without any cointegration) and the null hypothesis of *r* ≤ 1 (with cointegration). In the Johansen test, when the value for *r* ≤ 1 is greater than the confidence level value, there is cointegration. Table 4 shows the test results, which confirm that there is cointegration in both the regression of the indices and their respective growth rate regression.

According to Alexander [30], when there is cointegration between two time series a causal Granger-type relationship will also exist. Although cointegration is not required to indicate the presence of this relationship—which may reflect common characteristics between the series—the inverse is true, i.e., the presence of a causal relationship suggests that there is cointegration between the two time series.

To identify a causality relationship between the confidence index and the stock price, we conduct the Granger causality test with up to three discrepancies, for two hypotheses:

1. *H*_0_: Stock Price causes Confidence Index
2. *H*_1_: Confidence Index causes Stock Price

Table 5 shows the Granger test results for both hypotheses. Note that there is a causality relationship *from the stock price to the confidence index* but not from the confidence index to the stock price. In addition, there is a strong causality relationship (*R*^2^ close to 1) between both hypotheses, and the causality relationship remains in all of the discrepancies tested. A similar result can also be found in the analysis of the causality relationship *from the growth rate of the stock price to the growth rate of the confidence index*, as can be seen in Table 6.

**Table 5:** Results of the Granger test for the causality relationship analysis.

One discrepancy			Two discrepancies			Three discrepancies		
F-test	p-value	*R*^2^	F-test	p-value	*R*^2^	F-test	p-value	*R*^2^
**Price causes confidence**								
4.398977	0.0360106	1	3.948997	0.01933424	1	2.561213	0.05314514	1
**Confidence causes price**								
0.301555	0.5829337	1	0.126766	0.8809426	1	0.2289108	0.876315	1

**Table 6:** Results of the Granger test for the causality relationship analysis between the growth rate of confidence and the growth rate of price.

One discrepancy			Two discrepancies			Three discrepancies		
F-test	p-value	*R*^2^	F-test	p-value	*R*^2^	F-test	p-value	*R*^2^
**Price growth causes confidence growth**								
3.175428	0.0837517	1	0.063755	0.9382357	1	2.550253	0.05396217	1
**Confidence growth causes price growth**								
0.0096450	0.9217715	1	0.149433	0.8612009	1	0.9564453	0.412321	1

### Comparing our model with the S&P 500 and its respective confidence index

**Fig 11:**
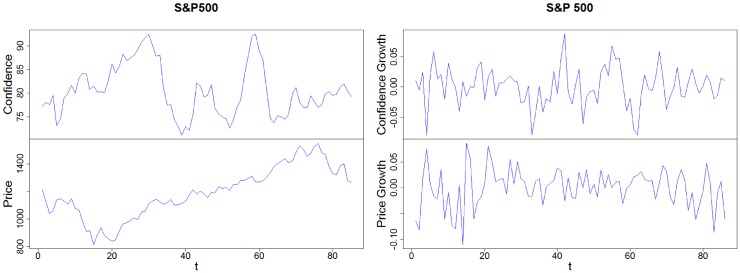
Time series illustrating the confidence index and the stock price for the S&P 500 (on the left) and the growth rate of the indices (on the right). Source: Own creation.

To estimate the robustness of our model, we compare its results in two cases: (i) the S&P 500 index and the stock market confidence indexes calculated by the Yale School of Management (data provided by the International Center for Finance, Yale School of Management, Yale University, USA. The monthly data refer to the period from July 2001 to August 2008. The period after August 2008 was not considered as the confidence index in the stock market strangely increased despite a major financial crisis that started in September of 2008, with the Lehman Brothers’ bankruptcy.), and (ii) the growth rate of both the S&P 500 and its confidence index. Fig 11 shows the time evolution with the confidence index (*C*_*t*_) and values for the S&P 500 index on the left and its respective growth rate data for the same variables on the right.

First we check the stationarity of the confidence series for the Yale confidence index and apply the four unit root tests. Most of the tests indicate that we cannot reject the null hypothesis (see Table 7). We then apply similar stationarity tests to the S&P 500, and obtain very similar results, suggesting that there is non-stationarity in the S&P 500 and in its corresponding Yale confidence indices.

**Table 7:** Unit root tests for the indexes.

	Confidence Yale	S&P 500
**LB Test** **(p < 0.05)**	p = 0.0001 (TRUE)	p = 0.0001 (TRUE)
**PP Test** **(p < 0.05)**	p = 0.01 (TRUE)	p = 0.01 (TRUE)
**ADF *τ* critical values** **1% 5% 10%** **-2.58 -1.95 -1.62**	*τ* = -0.06176 (FALSE)	*τ* = -0.25462 (TRUE)
**KPSS *τ* critical values** **1% 2.5% 5% 10%** **0.216 0.176 0.146 0.119**	*τ* = 0.3126 (TRUE)	*τ* = 0.1308 (FALSE)

To test the results of our model, we next check the cointegration among the prices and their respective confidence indices. Once again we address the Engle-Granger approach (Engle & Granger, 1987) by analyzing the stationarity of the residuals of the regression between the S&P 500 index with its respective confidence levels.

To test the cointegration for the (i) S&P 500 price index with respect to its respective Yale confidence index, we use the most negative Dickey-Fuller (DF) value to select the regression formula. Because the DF value for the regression formula “confidence vs price” for the S&P 500 is −1.8234, and −1.9289 for the “price vs confidence”, we choose “the confidence index” to be the dependent variable and find that we can reject the null hypothesis within a 90% confidence level (p-value is 10%). Thus we can assume that the residual is stationary, suggesting that there is a cointegration between confidence levels and the S&P 500 index.

In the (ii) S&P 500 growth rate with respect to its corresponding Yale confidence growth rate case (see Table 8), the DF value of the regression formula “growth confidence vs growth price” is −5.8269, and for “price vs confidence” it is −6.8574. We choose the “confidence index” as the dependent variable and find that we can reject the null hypotesis within a 90% confidence level (p-value is 10%), indicating that the residual is stationary, and that there is cointegration between the Yale confidence growth rate and the S&P 500 growth rate.

**Table 8:** Unit root tests for the growth indexes.

	Growth of Confidence Yale	Growth of S&P 500
**LB Test (p < 0.05)**	p = 0.1656 (FALSE)	p = 0.0001 (TRUE)
**PP Test (p < 0.05)**	p = 0.01 (TRUE)	p = 0.01 (TRUE)
**ADF *τ* critical values** **1% 5% 10%** **-2.58 -1.95 -1.62**	*τ* = -7.6861 (TRUE)	*τ* = -4.10604 (TRUE)
**KPSS *τ* critical values** **1%, 2.5%, 5%, 10%** **0.216, 0.176, 0.146, 0.119**	*τ* = 0.2358 (TRUE)	*τ* = 0.1096 (FALSE)

In an alternative approach, we apply the Johansen test to both indices and find that there is cointegration in all cases: (i) between the S&P 500 and the Yale confidence indexes, and also (ii) between the growth rates for both indices (see Table 9).

**Table 9:** Johansen test for confidence x S&P 500.

	Value test for the regression formula:		Level of confidence		
	“confidence vs price”	“growth of confidence vs growth of price”	10%	5%	1%
S&P 500 x Confidence Yale					
*r* = 0	6.56	28.94	6.50	8.18	11.65
*r* ≤ 1	24.86	68.95	15.66	17.95	23.52

In order to determine causality relations, we apply the Granger tests to all of the series. Table 10 shows the Granger test result for these indices. The F-test values and p-value for “S&P 500 causes Confidence” indicate that there is a causality relationship, especially when two or three discrepancies are considered. In the reverse “Confidence causes S&P 500” case, the F-test values and p-value indicate that the relationship is nonexistent.

**Table 10:** Granger test results—causality relationship between the S&P 500 index and its respective confidence index.

One discrepancy			Two discrepancies			Three discrepancies		
F-test	p-value	*R*^2^	F-test	p-value	*R*^2^	F-test	p-value	*R*^2^
**S&P 500 causes Confidence**								
0.00072046	0.978651	1	2.324916	0.104365	1	1.194534	0.031750	1
**Confidence causes S&P 500**								
0.4351712	0.511289	1	0.430911	0.651416	1	0.664147	0.576614	1

Table 11 shows the result of the Granger test for the growth rates of these indices. The F-test values and p-value for “S&P 500 causes Confidence” indicate that there is a causality relationship (with *p*_*value*_ ≤ 0.1), especially when one or two discrepancies are considered. In the reverse “Confidence causes S&P 500” case, the F-test values and p-value indicate that the relationship is not significant. As far as we know, there are only two stock market confidence indexes: the one calculated by Yale University and the other by Prof. Tsutsui from Osaka University, Japan. Therefore, similarly to the S&P 500 and its confidence indices, we repeated the same procedure for the Japanese stock market Nikkei index and its confidence level. We find that the Nikkei index is non stationary. However, a stationary behavior was found in its corresponding Osaka confidence index for the period of time that we were interested in. So, this result did not allow us to proceed with a proper analysis with the methods we used to check the cointegration and the causality relation between the Japanese indices.

**Table 11:** Granger test results—causality relationship between the growth rates of the S&P 500 index and its respective confidence index.

One discrepancy			Two discrepancies			Three discrepancies		
F-test	P–value	*R*^2^	F-test	P -value	*R*^2^	F-test	P–value	*R*^2^
**S&P 500 causes Confidence**								
3.276768	0.07307387	1	19.51692	6.3521e-08	1	7.40147	0.0001567	1
**Confidence causes S&P 500**								
1.293229	0.2579939	1	16.56494	5.68698-07	1	3.196363	0.2667843	1

## Final considerations

Using an agent based model, we first analyze the interactions between agents using different trading strategies. We find that behavioral heterogeneity causes asset prices to be significantly more volatile than fundamental stock values. Next, we use agent-based modeling to analyze how both excess trader confidence and low trader confidence affects stock market trader decision-making, stock price dynamics, and rates of return and how agent confidence levels change over time. We find that agent decisions are strongly affected by agent confidence level, and that agent overconfidence strongly contributes to bubble formation.

We also find that the price series and confidence levels generated by our model, as well as their growth rates, are non-stationary and cointegrated. We use the Granger test to identify causality relationships between the two variables and find that price affects confidence level, but that confidence level does not affect price. The same results are obtained for their growth rates.

To compare our model with actual data, we examine the S&P 500 index and its respective confidence levels. As in our model, Engle-Granger and Johansen tests indicate that there is cointegration between stock prices and stock market confidence indices, and between price growth and confidence growth rate. Besides, the Granger causality test indicates that price or its growth rate affects confidence and its growth rate. Therefore, we can assume it supports the predictions of our agent model and we thus conclude that when we use our proposed agent model to analyze historic price indices we are able to usefully estimate future market behavior.

## Supporting information

S1 Appendix A1 Table, Values attributed to general parameters. A2 Table, Descriptive Statistics (Agents 100% Fundamentalists). A1 Fig, Evolution of the Stock Rate of Return (Agents 100% Fundamentalists). A2 Fig, Shapiro -Wilk Normality Test Stock Rate of Return (Agents 100% Fundamentalists).
(PDF)
