# Supplementary material for: Confidence and self-attribution bias in an artificial stock market
Source: PLoS One. 2017 Feb 23;12(2):e0172258. doi: 10.1371/journal.pone.0172258 (PMC5322910; doi:10.1371/journal.pone.0172258)
Supplement: S1 Appendix — A1 Table, Values attributed to general parameters. A2 Table, Descriptive Statistics (Agents 100% Fundamentalists). A1 Fig, Evolution of the Stock Rate of Return (Agents 100% Fundamentalists). A2 Fig, Shapiro -Wilk Normality Test Stock Rate of Return (Agents 100% Fundamentalists). (PDF) [file pone.0172258.s001.pdf]

Supporting Information

S1 Appendix

Table A1. Values attributed to general parameters.

Parameters	Values
Number of Agents	100
$\bar{d}$	4
d_{t-1}	4
P	0.95
$mean \epsilon_t$	0
var ϵ_t	0.0742
p_{t-1}	20
R	0.10
B	2000
Λ	0.5
$W_{t-1,i}$	100
$E_{i,t-1}(p_{t+1} + d_{t+1})$	22
$\sigma^2_{i,t-1,p+d}$	4
$x_{i,t-1}$	1
G	15
K	0.25
Θ	0.01
$oc_{i,t-1}$	1
$\bar{a}$	0.95
$\bar{b}$	1.05

Table A2. Descriptive Statistics (Agents 100% Fundamentalists).

	Stock Price	Return
**Mean**	19.1530	0.2082
**Median**	19.1331	0.2080
**St.Deviation**	0.4307	0.0187
**Kurtosis**	0.0591	1.0917
**Skewness**	0.2951	0.0958

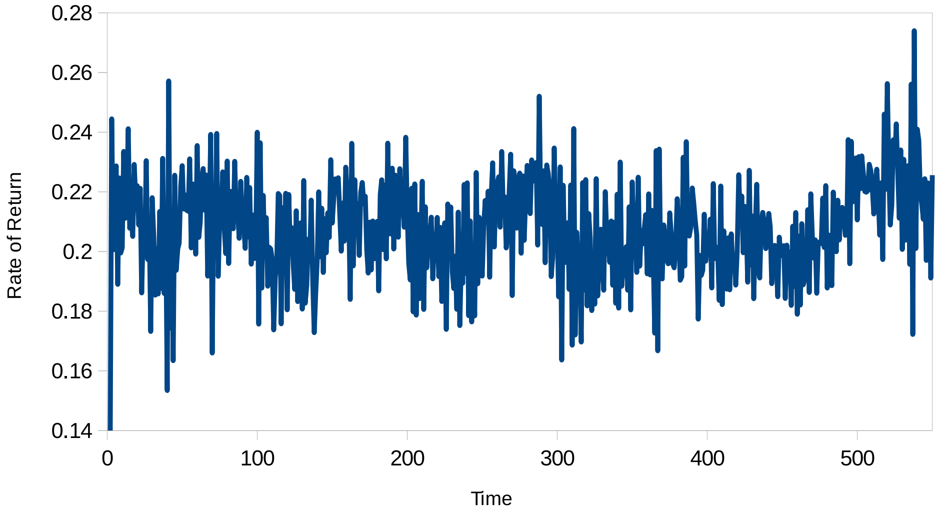

Figure A1. Evolution of the Stock Rate of Return (Agents 100% Fundamentalists).

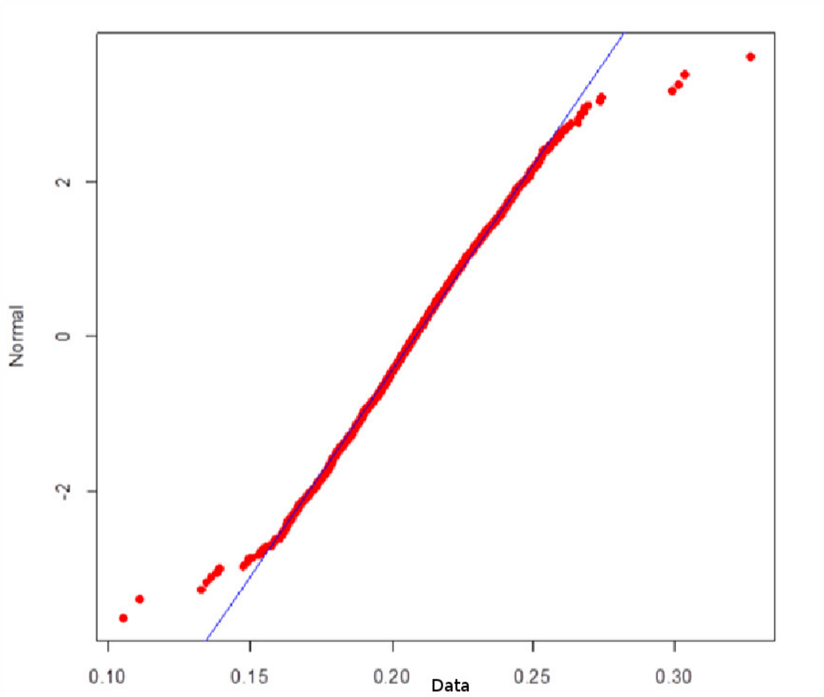

Figure A2. Shapiro -Wilk Normality Test Stock Rate of Return (Agents 100% Fundamentalists).
